# Metabolic syndrome components and acute pancreatitis: a case–control study in China

**DOI:** 10.1186/s12876-020-01579-3

**Published:** 2021-01-06

**Authors:** Zhemin Shen, Xueqiao Wang, Zili Zhen, Yao Wang, Peilong Sun

**Affiliations:** 1 Department of General Surgery, Jinshan Hospital, Fudan University, Shanghai, China; 2 Department of Otorhinolaryngology Head and Neck Surgery, Jinshan Hospital, Fudan University, Shanghai, China; 3 Department of Orthopedics, Jinshan Hospital, Fudan University, Shanghai, China

**Keywords:** Acute pancreatitis, Metabolic syndrome, Hyperglycaemia, Hyperlipidaemia, Obesity, Hypertension

## Abstract

**Background:**

Acute pancreatitis (AP) is a common inflammatory disorder of the pancreas. Recent evidence has shown that metabolic syndrome is positively correlated with the severity of AP. However, only a few studies have revealed the relationship between metabolic syndrome and the occurrence of AP. We therefore elucidated the association between metabolic syndrome and the occurrence of AP.

**Methods:**

A hospital-based case–control study was conducted. A total of 705 patients admitted to our hospital from January 2016 to December 2018 were included in the study. Subjects were divided into case and control groups according to their diagnosis: (1) According to the revised Atlanta classification from 2012, patients diagnosed with AP were enrolled in the case group. (2) Patients without a history of AP or any disease related to metabolic syndrome were allocated into the control group. Controls were matched to cases individually by sex and age (control/case ratio = 1).

**Results:**

The incidence rate of metabolic syndrome in AP patients was 30.9%, which was more frequent than that in controls (13.2%) (OR 2.837; 95% CI 1.873–4.298, *p* < 0.001). In the multivariate regression analysis, a history of smoking or alcohol consumption and biliary stones were significantly associated with AP (OR 2.441; 95% CI 1.865–5.172, *p* < 0.001; OR 1.777; 95% CI 1.060–2.977, *p* = 0.029; OR 28.995; 95% CI 13.253–63.435, *p* < 0.001). In addition, the occurrence of AP was significantly associated with total cholesterol (TC) (OR 1.992; 95% CI 1.246–3.183, *p* = 0.004), triglyceride (TG) (OR 2.134; 95% CI 1.403–3.245, *p* < 0.001), hyperglycaemia (OR 2.261; 95% CI 1.367–3.742, *p* = 0.001), and apolipoprotein A (Apo A) (OR 0.270; 95% CI 0.163–0.447, *p* < 0.001).

**Conclusions:**

Metabolic syndrome and its components were associated with AP occurrence.

## Background

Acute pancreatitis (AP) is a pancreatic inflammatory disorder that may cause life-threatening consequences due to severe inflammatory responses [1]. According to a survey, the annual incidence rate of AP has increased by 100% over the past several decades [2]. This has posed a great threat to human health and become one of the largest contributors to aggregate medical costs [3]. The main pathogenic causes of AP are biliary stones and alcohol consumption. Other factors, such as smoking and genetic factors, are also considered to be related to the occurrence of AP [3]. However, the pathogenic immune mechanism of AP remains elusive, and the potential factors related to the stimulus of inflammation are still under investigation.

Due to the huge economic development and social progresses made in China over past decades, the lifestyles and daily diets of Chinese people have changed substantially. In turn, this has led to the high prevalence of metabolic syndrome in China (27.9% in men and 26.8% in women) and has caused serious public health problems [4]. Metabolic syndrome is defined as four interconnected factors: hyperglycaemia, hyperlipidaemia (particularly increased triglycerides [TGs] and low high-density lipoprotein [HDL] cholesterol), obesity and hypertension. It is generally considered a risk factor for cardiovascular diseases [5]. Although there is still no universally accepted mechanism of metabolic syndrome [6], the best evidence suggests that the four components of metabolic syndrome may be intercorrelated with each other by sharing common pathophysiological processes. These processes mainly consist of insulin resistance, visceral adiposity, atherogenic dyslipidaemia and endothelial dysfunction [7]. In addition, given that the four components are more likely to appear together more than might be expected by chance, metabolic syndrome should thus be considered an overall concept. It can gradually lead patients to a proinflammatory state that is associated with a series of diseases, such as venous thrombosis and psoriasis [7–9]. Since it is well believed that there is an interrelation between metabolic syndrome and inflammation [10, 11], we suspect that metabolic syndrome may also have a significant impact on AP. Although some studies have demonstrated that metabolic syndrome is positively associated with the severity of AP [1, 12], there is still a lack of data showing the relationship between metabolic syndrome and the occurrence of AP in the Chinese population, and most of them involved mainly the single component of metabolic syndrome, such as obesity or hyperglycaemia, rather than as an overall concept [1, 13–16, 20]. Moreover, these studies were mainly conducted on western countries, which may differ from those in eastern populations. To address these issues, we examined relevant data to investigate (1) the association between metabolic syndrome and the occurrence of AP, (2) the association between each metabolic syndrome component and the occurrence of AP, (3) the association between the number of metabolic syndrome components and the occurrence of AP.

## Methods

### Ethical approval

The study was conducted according to the Declaration of Helsinki and approved by the institutional research board of Jinshan Hospital (IEC-2020-S21). Informed consent was waived.

### Study population

A hospital-based case–control study was conducted. A total of 705 patients (349 AP patients and 356 controls) admitted to our hospital from January 2016 to December 2018 were included in the study. According to the revised Atlanta classification from 2012 [17], AP was defined if at least two of the following three criteria were presented: acute attack of severe upper abdominal pain with or without radiating to the back; serum amylase or lipase elevation at least three times above the upper limit of normal level; and typical acute pancreatitis imaging found on computed tomography (CT) scan. Only patients with a first attack of AP were included, while patients with relapse of AP or chronic pancreatitis were excluded. The controls were selected from patients admitted to department of anorectal surgery, department of otorhinolaryngology head and neck surgery and department of orthopedics. Their diagnosis mainly consisted of haemorrhoids, sudden sensorineural hearing loss, tonsil hypertrophy, epiglottic cyst and disc herniation. The controls were frequency matched to cases by sex and exact age. Control subjects were excluded if they had AP history. Participants who had histories of any cardiovascular disease (except hypertension) or psoriasis were not enrolled in the two groups. Last, subjects were excluded if they were younger than 18 years, pregnant, underwent surgery within 1 month or had a cancer history.

All patients admitted were given standard medical treatment. In AP group, patients received treatment including fasting, early fluid resuscitation, analgesia, and nutritional support. In non-AP group, patients mainly received pre-operation examinations such as ECG (Electrocardiogram) and chest X-Ray after admission since most of the patients were about to undergo surgery in the next few days.

### Clinical data collection

Data were collected by researchers who were not informed of the detailed information of the study until collection was completed. The collected data were from computer-based case reports and were checked by another two researchers to ensure that there were no inconsistencies or errors. The following data of patients were collected: sex; age; smoking history; alcohol drinking history (> 14 drinks/week in women or > 21 drinks/week in men); biliary stones history; history of hepatitis B or hepatitis C; body mass index (BMI); blood pressure; and laboratory tests, including total cholesterol (TC), TG, HDL, low-density lipoprotein (LDL), apolipoprotein A (Apo A), apolipoprotein B (Apo B), and fasting plasma glucose (FPG). BMI was calculated as body weight (kg) divided by square of the height (m). Blood pressure was measured by a mercury sphygmomanometer when patients were in a supine position and after 20 min of rest. Blood samples were collected from the median cubital vein of each patient after 8 h of fasting. Fasting venous blood was collected in polystyrene tubes and rapidly transmitted to the laboratory to ensure the accuracy of our indexes.

### Metabolic syndrome components

The diagnostic criteria for metabolic syndrome were defined according to National Cholesterol Education Program Adult Treatment Panel III (NCEP/ATP-III) [18] and modified by the Asia–Pacific criteria [19]. The criteria include (1) hypertension (blood pressure ≥ 130/85 mmHg or ongoing anti-hypertensive treatment), (2) hyperlipidaemia (TG ≥ 150 mg/dL or HDL ≤ 40 mg/dL in men and 50 mg/dL in women or ongoing anti-lipidaemic treatment), (3) hyperglycaemia (FPG ≥ 110 mg/dL, previously physician-diagnosed type 2 diabetes mellitus [T2DM] or ongoing antidiabetic treatment) and (4) obesity (BMI ≥ 25 kg/m^2^ or waist circumference ≥ 90 cm for men and 80 cm for women).

### Statistical analysis

Quantitative data were expressed with mean ± standard deviation (SD) or median with 25^th^ and 75^th^ percentiles as appropriate. For normal distributed quantitative data, independent sample t-test was used to compare the differences between the two groups. For non-parametric quantitative data, Mann–Whitney test was used to detect the statistical significance. Qualitative data were expressed in number (n) and percentage (%). Fisher’s exact test or chi-square test was used to compare the differences. Univariate logistic regression analysis was used to preliminarily assess whether these individual variables were predictive factors of AP occurrence. Odds ratios (ORs) and corresponding 95% confidence intervals (CIs) were calculated. Univariate and multivariate logistic regression were used to further test the OR value. The accuracy of each marker in predicting the occurrence of AP was assessed by using receiver operating characteristic (ROC) curves. A *P* value < 0.05 was defined as statistically significant. IBM SPSS v22 (SPSS, Chicago, Illinois, USA) and MedCalc statistical software packages, version 10 (MedCalc, Mariakerke, Belgium), were used for statistical analysis.

## Results

### Characteristics of study subjects

Based on the inclusion and exclusion criteria, 705 subjects, including 349 AP patients and 356 non-AP patients, were enrolled in our study. Table 1 shows the characteristics of the subjects. No significant differences between AP patients and non-AP patients were found with respect to age (*p* = 0.989), gender (*p* = 0.923), hepatitis C (*p* = 0.349) and Apo B (*p* = 0.198). However, we noted that AP patients were significantly associated with cigarette smoking (*p* < 0.001), alcohol consumption (*p* < 0.001), biliary stones history (*p* < 0.001), hepatitis B (*p* = 0.049), and BMI (*p* < 0.001) and TC (*p* < 0.001), TG (*p* < 0.001), HDL (*p* < 0.001), LDL (*p* = 0.030), Apo A (*p* < 0.001), and FPG (*p* < 0.001) than controls. In patients with diabetes mellitus, 27 patients (79.4%) received anti-diabetic therapy in control group, while 59 patients (64.8%) received treatment in case group. There were no significant differences (*p* = 0.118) between the two groups regarding the anti-diabetic therapy.

**Table 1:** Basic characteristics

	Non-AP patients (n = 356)	AP patients (n = 349)	*p*
Mean age, years (SD)	51.2 ± 15.9	51.2 ± 15.9	0.989
Gender, n (%)			
Female	140 (39.3)	136 (39.0)	
Male	216 (60.7)	213 (61.0)	0.923
Smoking history, n (%)	62 (17.4)	125 (35.8)	< 0.001
Alcohol drinking history, n (%)	53 (14.9)	101 (28.9)	< 0.001
Biliary stones, n (%)	8 (2.3)	101 (28.9)	< 0.001
Hepatitis B, n (%)	17 (4.8)	7 (2.0)	0.049
Hepatitis C, n (%)	3 (0.8)	1 (0.3)	0.349
BMI, kg/m^2^ (IQR)	23.8 ± 3.5	24.8 ± 3.7	< 0.001
TC, mg/dL (IQR)	178.0 (153.7, 209.3)	180.3 (145.6, 235.1)	< 0.001
TG, mg/dL (IQR)	101.8 (77.0, 157.5)	131.9 (81.4, 349.6)	< 0.001
HDL, mg/dL (IQR)	99.1 (86.7, 117.7)	90.3 (74.3, 112.4)	< 0.001
LDL, mg/dL (IQR)	269.9 (224.8, 318.6)	258.4 (198.2, 236.3)	0.030
Apo A, g/L (IQR)	1.2 (1.1, 1.4)	1.1 (0.9, 1.3)	< 0.001
Apo B, g/L (IQR)	0.9 (0.7, 1.0)	0.8 (0.7, 1.0)	0.198
FPG, mg/dL (IQR)	97.8 (89.4, 112.1)	132.8 (107.4, 172.7)	< 0.001
Anti-diabetic therapy, n (%)	27 (79.4)	59 (64.8)	0.118

Data were numbers and percentages, or median (25th, 75th percentile), as appropriate

n number, *IQR* interquartile range, *BMI* body mass index (calculated as weight in kilograms divided by height in meters squared), *TC* total cholesterol, *TG* triglyceride, *HDL* high density lipoprotein, *LDL* low density lipoprotein, *Apo A* apolipoprotein A, *Apo B* apolipoprotein B, *FPG* fasting plasma glucose

SI conversion factors: TC: 1 mg/dL = 0.0259 mmol/L; HDL, LDL, TG: 1 mg/dL = 0.0113 mmol/L; FPG: 1 mg/dL = 0.0555 mmol/L

### Effect of metabolic syndrome components on AP

**Fig. 1:**
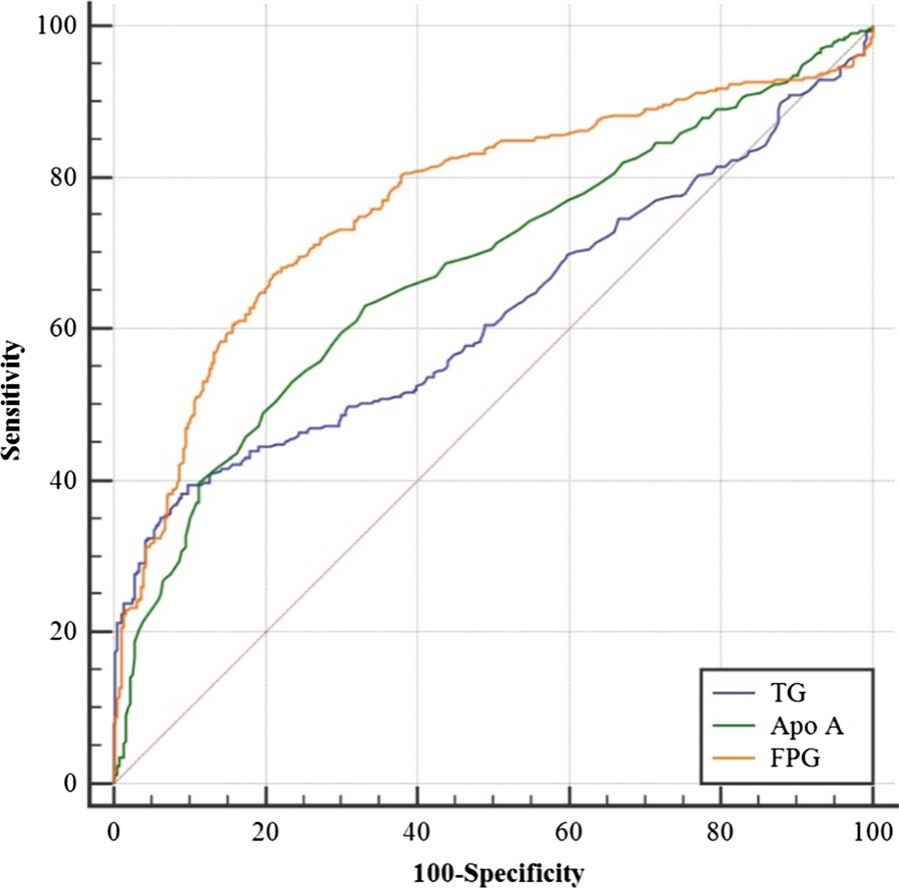
Receiver operating characteristic (ROC) curve analysis for predicting the occurrence of AP by TG, Apo A and FPG in the estimation cohorts. *AP* acute pancreatitis, *TG* triglyceride, *Apo A* apolipoprotein A, *FPG* fasting plasma glucose

In univariate analysis, both of increased values of TG and decreased values of HDL were associated with the occurrence of AP (OR 2.313; 95% CI 1.690–3.167, *p* < 0.001; OR 0.582; 95% CI 0.431–0.786, p < 0.001) (shown in Table 2). Additionally, significant differences were observed between the two groups in patients with obesity, hyperglycaemia and hypertension(OR 1.608; 95% CI 1.186–2.181, *p* = 0.002; OR 3.209; 95% CI 2.112–4.876, p < 0.001; OR 1.473; 95% CI 1.089–1.991, p = 0.012). In multivariate logistic regression models, after adjustment for smoking and alcohol drinking history, biliary stones history, Apo A and the components of metabolic syndrome, the results revealed that AP was associated with smoking history; alcohol consumption history; biliary stones history; elevated levels of TC, TG; hyperglycaemia and lower values of Apo A (OR 2.441; 95% CI 1.865–5.172, *p* < 0.001; OR 1.777; 95% CI 1.060–2.977, *p* = 0.029; OR 28.995; 95% CI 13.253–63.435, *p* < 0.001; OR 1.992; 95% CI 1.246–3.183, *p* = 0.004; OR 2.134; 95% CI 1.403–3.245, *p* < 0.001; OR 2.261; 95% CI 1.367–3.742, *p* = 0.001 and OR 0.270; 95% CI 0.163–0.447, *p* < 0.001, respectively). However, obesity was not observed to be associated with the occurrence of AP (*p* = 0.246) (shown in Table 3). After adjusting for smoking and alcohol drinking history, biliary stones, the prevalence of metabolic syndrome was more common in AP patients (30.9%) than in those without AP (13.2%) (OR 2.837; 95% CI 1.873–4.298, *p* < 0.001) (shown in Table 4). As shown in Table 5 and Fig. 1, for all AP patients, increased values of TG and low Apo A and FPG predicted AP with statistical significance (*p* < 0.001, *p* < 0.001, *p* < 0.001); their AUCs were 0.620, 0.679 and 0.767, respectively. Among the three indicators, FPG had the best sensitivity (67.54%), and TG had the best specificity (90.17%) when the indicators were at their best cut-off values (shown in Table 5).

**Table 2:** Univariate analysis examining the components of the metabolic syndrome

	Non-AP patients (n = 356)	AP patients (n = 349)	OR (95% CI)	*p*
TG ≥ 150 mg/dL, n (%)	97 (27.2)	162 (46.4)	2.313 (1.690–3.167)	< 0.001
HDL ≥ 40 mg/dL (M) or ≥ 50 mg/dL (F), n (%)	183 (51.4)	133 (38.1)	0.582 (0.431–0.786)	< 0.001
Obesity, n (%)	120 (33.7)	157 (45.0)	1.608 (1.186–2.181)	0.002
Hyperglycaemia, n (%)	37 (10.4)	93 (26.7)	3.209 (2.112–4.876)	< 0.001
Hypertension, n (%)	129 (36.2)	159 (45.6)	1.473 (1.089–1.991)	0.012

*OR* odds ratio, *TG* triglyceride, *HDL* high density lipoprotein

**SI conversion factors**: HDL, TG: 1 mg/dL = 0.0113 mmol/L

**Table 3:** Multivariate analysis examining the components of the metabolic syndrome

	Non-AP patients (n = 356)	AP patients (n = 349)	OR (95% CI)	*p*
Smoking, n (%)	62 (17.4)	125 (35.8)	2.441 (1.865–5.172)	< 0.001
Alcohol drinking, n (%)	53 (14.9)	101 (28.9)	1.777 (1.060–2.977)	0.029
Biliary stone, n (%)	8 (2.3)	101 (28.9)	28.995 (13.253–63.435)	< 0.001
Hepatitis B, n (%)	17 (4.8)	7 (2.0)	0.439 (0.145–1.327)	0.145
Obesity, n (%)	120 (33.7)	157 (45.0)	1.258 (0.854–1.855)	0.246
TC ≥ 220 mg/dL, n (%)	61 (17.1)	106 (30.4)	1.992 (1.246–3.183)	0.004
TG ≥ 150 mg/dL, n (%)	97 (27.2)	162 (46.4)	2.134 (1.403–3.245)	< 0.001
HDL ≥ 40 mg/dL (M) or ≥ 50 mg/dL (F), n (%)	183 (51.4)	133 (38.1)	0.784 (0.522–1.176)	0.240
Apo A ≥ 1 g/L, n (%)	321 (90.2)	230 (65.9)	0.270 (0.163–0.447)	< 0.001
Hyperglycaemia, n (%)	37 (10.4)	93 (26.7)	2.261 (1.367–3.742)	0.001
Hypertension, n (%)	129 (36.2)	159 (45.6)	1.189 (0.817–1.732)	0.366

*OR* odds ratio, *TC* total cholesterol, *TG* triglyceride, *HDL* high density lipoprotein, *Apo A* apolipoprotein A

**SI conversion factors**: TC: 1 mg/dL = 0.0259 mmol/L; HDL, TG: 1 mg/dL = 0.0113 mmol/L

**Table 4:** Association between the metabolic syndrome and AP

	Prevalence of metabolic syndrome	OR (95% CI)	*p*
AP patients, n (%)	108 (30.9)	2.837 (1.873–4.298)	< 0.001
Non-AP patients, n (%)	47 (13.2)		

*OR* odds ratio, *AP* acute pancreatitis, *CI* confidence interval

**Table 5:** Identification of TG, Apo A and FPG in the patients with AP

Variable	AUC	*p*	Cut-off	Sensitivity (%)	Specificity (%)	+ LR	− LR
TG, mg/dL	0.620	< 0.001	218.6	39.08	90.17	3.98	0.68
Apo A, g/L	0.679	< 0.001	1.15	63.16	66.85	1.91	0.55
FPG, mg/dL	0.767	< 0.001	115.1	67.54	78.93	3.21	0.41

*AUC* area under curve, *LR* likelihood ratio, *TG* triglyceride, *Apo A* apolipoprotein A, *FPG* fasting plasma glucose

**SI conversion factors**: TG: 1 mg/dL = 0.0113 mmol/L; FPG: 1 mg/dL = 0.0555 mmol/L

### Association between the number of metabolic syndrome components and AP

**Fig. 2:**
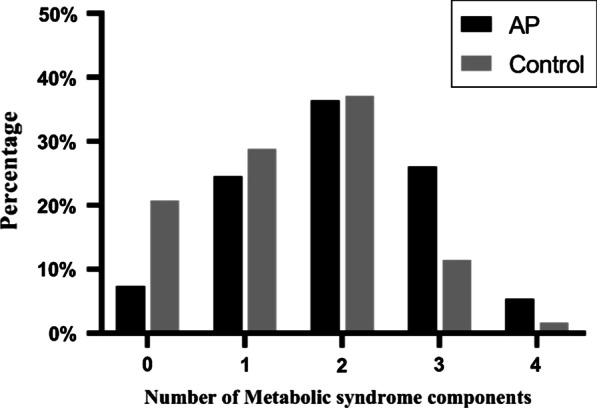
The number of metabolic syndrome components in relation to the occurrence of AP. *AP* acute pancreatitis

As shown in Fig. 2, the incidence rates of AP obviously increased when there were more than three components of metabolic syndrome present. Moreover, the incidence rates of AP significantly declined when there were no components of metabolic syndrome present.

## Discussion

To our knowledge, this is the first case–control study demonstrating the relationship between metabolic syndrome and the occurrence of AP in an Asian population. Our results showed that metabolic syndrome was positively associated with the occurrence of AP. Among components of metabolic syndromes, we revealed that hyperglycaemia and hyperlipidaemia were independently associated with AP.

Hyperglycaemia has been considered to be associated with AP for decades [20]. However, only a few studies have shown a higher incidence rate of AP among patients with hyperglycaemia [13–16]. Our results revealed that hyperglycaemia had positively correlation with AP (OR 2.261; 95% CI 1.367–3.742, *p* = 0.001). Although the exact mechanism between hyperglycaemia and AP remains unclear, several underlying biological theories have been proposed. High plasma glucose enhances mitochondrial oxidative stress by promoting the production of reactive oxygen species (ROS) and lipid oxidation through cytosolic Ca^2+^ accumulation [21–23]. Furthermore, owing to the dysfunction of beta cells and resultant hyperinsulinaemia, beta cells may lose their sensitivity to the inhibitory hormone somatostatin, which may be an important factor in inducing AP [24]. Moreover, insulin resistance, as a crucial pathophysiological factor of hyperglycaemia, has been reported to be involved in AP development. Various proinflammatory factors or cytokines are activated due to insulin resistance, including nuclear factor-kappaB (NF κB), tumour necrosis factor-alpha (TNF-α), amylin and interleukin-6, and these proinflammatory factors may be responsible for the initiation and progression of AP [25–29].

It is well known that hypertriglyceridaemia is associated with the morbidity and mortality of AP [30–32]. Our study showed that TG was associated with AP. Furthermore, we found that TC and Apo A values were also correlated with the occurrence of AP, which demonstrated that atherogenic lipid profiles also participated in the development of AP. Triantafilou M et al. reported that cholesterol may trigger inflammatory responses that could lead to chronic inflammation and insulin resistance via Toll-like receptor 4 (TLR4) and ultimately cause lysosomal damage, ROS generation and proinflammatory cytokine secretion [33, 34]. This theory may explain why acinar injury is induced by hypercholesterolaemia through the inflammatory response. Furthermore, as a lipoprotein, HDL often opposes cholesterol accumulation and reduces inflammation by the ATP-binding cassette transporter A1 (ABCA1) pathway [35]. This ability may be impaired if Apo A is oxidized by macrophage myeloperoxidase (MPO). Shao B et al. reported impaired function of HDL and Apo A in patients with atherosclerosis [36], which may be similar in AP. However, our results only showed that the value of Apo A was negatively associated with the occurrence of AP, while we did not observe an association between HDL and AP. Based on our present study, it is hard to tease out the exact role of HDL in AP. In future studies, it will be important to determine the exact function that HDL and Apo A exert in AP development.

Currently, obesity is considered a global pandemic that poses a great threat to human health. Some studies have shown that obesity is positively correlated with the severity of AP [12, 37, 38]. However, due to the paucity of studies between obesity and the incidence of AP, their relationship has been a matter of dispute. Blomgren et al. found that the crude risk ratio (RR) between obesity and the occurrence of AP was 1.8 (95% CI 1.3–2.6), while the authors did not display the results after adjustment [39]. In contrast to previous beliefs, our current study did not find an association between obesity and AP (*p* = 0.246), which is consistent with the findings of a recent large-scale prospective cohort study (RR = 1.02, 95% CI 0.68–1.53) [40]. In an animal model, obesity was shown to be related to an inflammatory status by secreting proinflammatory cytokines such as TNF-α and interleukin-6 [41]. Moreover, the level of adiponectin, an anti-inflammatory cytokine, is reduced in an obese environment. Therefore, combined with previous data and our results [12], we suppose that obesity may aggravate the severity of AP but is not enough to initiate the development of AP without other risk factors in a non-AP person.

Recently, visceral obesity, a subtype of obesity, is considered as a metabolically active status. Previous study suggested that occurrence of biliary pancreatitis was positively correlated with intrapancreatic fat. It may be caused by saturation of cholesterol in bile, which could consequently induce gallstone formation [42]. Moreover, visceral fat was also associated with the severity of AP [43]. The level of adiponectin would paradoxically decrease due to excess viscera fat, which would subsequently increase the secretion of proinflammatory cytokines such as TNF-α and interleukin-6, and thus amplify the inflammatory response [44, 45]. Meanwhile, severe lipo-toxicity caused by visceral obesity could also induce the organ failure in AP patients [46]. However, our data did not show the significant difference between obesity and AP since only general obesity was measured in the current study. The different clinical outcomes that induced by general obesity and visceral obesity may be a kind of obesity paradox, even though its mechanism is still unclear. Therefore, future studies need to focus on this obesity paradox.

The limitations of our study are as follows: (1) Our study is a retrospective hospital-based case–control study, which can only assess the association. Prospective studies are needed to evaluate the causation between metabolic syndrome and AP in the future. (2) The visceral adiposity was not assessed and obesity was only measured by BMI in the current study. (3) Although we found a strong association between metabolic syndrome components and AP, we did not evaluate the effect of the treatment of each component on individuals, which may have an impact on the results. The major strengths are as follows: (1) This is the first study to illustrate the association between metabolic syndrome and the development of AP in an Asian population. (2) Given the completeness of behavioural and epidemiologic data, we can ensure the accuracy of clinical diagnosis and high quality of data and minimize recall bias.

## Conclusions

The study revealed that patients with metabolic syndrome were associated with high incidence rates of AP. Regarding metabolic syndrome components, high levels of TC, TG, and FPG and low values of Apo A were independently associated with the development of AP. Since metabolic syndrome can be treated by lifestyle alterations or pharmacological treatment, we are looking forward to exploring this potential method to impede the increasing incidence rates of AP.

## Data Availability

The datasets generated during the current study are not publicly available due to confidentiality of human subjects but are available from the corresponding author on reasonable request.

## Abbreviations

AP: Acute pancreatitis
CT: Computed tomography
ECG: Electrocardiogram
BMI: Body mass index
TC: Total cholesterol
TG: Triglyceride
HDL: High density lipoprotein
LDL: Low density lipoprotein
Apo A: Apolipoprotein A
Apo B: Apolipoprotein B
FPG: Fasting plasma glucose
T2DM: Type 2 diabetes mellitus
OR: Odds ratio
CI: Confidence interval
AUC: Area under curve
LR: Likelihood ratio
ROS: Reactive oxygen species
NF-κB: Nuclear factor-kappaB
TNF-α: Tumour necrosis factor-alpha
TLR4: Toll-like receptor 4
ABCA1: ATP-binding cassette transporter A1
MPO: Macrophage myeloperoxidase
RR: Risk ratio
ROC: Receiver operating characteristic
